# Assessment of Universal Healthcare Coverage in a District of North India: A Rapid Cross-Sectional Survey Using Tablet Computers

**DOI:** 10.1371/journal.pone.0157831

**Published:** 2016-06-28

**Authors:** Tarundeep Singh, Pritam Roy, Limalemla Jamir, Saurav Gupta, Navpreet Kaur, D. K. Jain, Rajesh Kumar

**Affiliations:** 1 Department of Community Medicine & School of Public Health, Post Graduate Institute of Medical Education and Research, Chandigarh 160 012, India; 2 Center for Development of Advanced Computing, Mohali, Punjab, India; UNAIDS, GUYANA

## Abstract

**Objective:**

A rapid survey was carried out in Shaheed Bhagat Singh Nagar District of Punjab state in India to ascertain health seeking behavior and out-of-pocket health expenditures.

**Methods:**

Using multistage cluster sampling design, 1,008 households (28 clusters x 36 households in each cluster) were selected proportionately from urban and rural areas. Households were selected through a house-to-house survey during April and May 2014 whose members had (a) experienced illness in the past 30 days, (b) had illness lasting longer than 30 days, (c) were hospitalized in the past 365 days, or (d) had women who were currently pregnant or experienced childbirth in the past two years. In these selected households, trained investigators, using a tablet computer-based structured questionnaire, enquired about the socio-demographics, nature of illness, source of healthcare, and healthcare and household expenditure. The data was transmitted daily to a central server using wireless communication network. Mean healthcare expenditures were computed for various health conditions. Catastrophic healthcare expenditure was defined as more than 10% of the total annual household expenditure on healthcare. Chi square test for trend was used to compare catastrophic expenditures on hospitalization between households classified into expenditure quartiles.

**Results:**

The mean monthly household expenditure was 15,029 Indian Rupees (USD 188.2). Nearly 14.2% of the household expenditure was on healthcare. Fever, respiratory tract diseases, gastrointestinal diseases were the common acute illnesses, while heart disease, diabetes mellitus, and respiratory diseases were the more common chronic diseases. Hospitalizations were mainly due to cardiovascular diseases, gastrointestinal problems, and accidents. Only 17%, 18%, 20% and 31% of the healthcare for acute illnesses, chronic illnesses, hospitalizations and childbirth was sought in the government health facilities. Average expenditure in government health facilities was 16.6% less for acute care, 15% less for hospitalization and 50% less for childbirth than in the private healthcare facilities. Out-of-pocket expenditure was mostly on medicines followed by diagnostic and laboratory tests. Among households experiencing hospitalization, 56.5% had incurred catastrophic expenditures, which was significantly higher in the poorest compared to richest household expenditure quartile (p <0.002).

**Conclusions:**

Expenditure on healthcare remains high in Punjab state of India. Efforts to increase utilization of the public sector could decrease out-of-pocket healthcare expenditure.

## Introduction

Universal Healthcare (UHC) focuses on inclusive and unified health services based on primary healthcare delivered in a comprehensive and integrated manner[1]. A summary measure of UHC is “the percentage of those in need of healthcare, who are able to access appropriate health care which is effective, without financial hardship”. Related measures may include the illness and healthcare utilization pattern in the population, expenditure on healthcare, catastrophic expenditure on healthcare etc^1^. In India, the National Sample Survey Organization (NSSO) collects this type of information at an interval of about ten years at state and national level [2]. Information on risk factors, disease burden, and utilization of healthcare services is also obtained though National Family Health Survey (NFHS), Global Adult Tobacco Survey (GATS), and the District Level Household and Facility Surveys [3–5]. These large scale surveys are usually expensive, logistically difficult, and time consuming.

In situations where program data are not very regularly available, governments and other agencies usually face difficulties in tracking progress towards the set goals. Rapid surveys can provide information on key indicators in a reliable manner for taking programmatic decisions in a variety of settings where resources and data are scarce. Such methodologies have been extensively used in estimating immunization coverage in developing countries and also to assess health needs of communities at local level in developed countries[6].

Reports from several countries suggest that data collection for rapid surveys on handheld computing devices has addressed the issues of cost, timeliness, and data quality in surveys [7]. Recently, Nepal and Nigeria have successfully used tablets to collect demographic and health data [8,9].

Since monitoring of UHC requires information at frequent intervals to facilitate need based decision making by the program managers, we conducted a rapid survey to measure pattern of illness, healthcare utilization and expenditure on healthcare in Shaheed Bhagat Singh (SBS) Nagar District of Punjab, India.

## Methodology

A rapid survey was conducted in SBS Nagar district of Punjab, India during April and May, 2014. This district was chosen because of high vacancy rates in the health workforce as compared to the other districts of Punjab. Government of Punjab has stated intention to boost the healthcare infrastructure in SBS Nagar to achieve the goals of UHC. Total population of the district in 2011 was 6,12,310. The district has 8 towns and 458 inhabited villages[10].

SBS Nagar district has a three tiered formal government healthcare system with a district and a sub-district hospital in the top tier, three community health centers (CHCs) in the middle tier, and 17 primary health centers (PHCs) and 47 subsidiary health centers (SHCs) forming the third tier. In addition, there are 95 sub-centers (SCs) covering remote rural areas which are staffed by Auxiliary Nurse Midwives. Private healthcare consists of 113 clinics, 53 secondary level and 5 tertiary level hospitals. Most villages also have informal unqualified medical practitioners. In 2012–2013, full antenatal care coverage was 28.8%, and 80.9% of the deliveries took place in healthcare institutions and 72.2% of children aged 12 to 23 months were fully immunized in this district [11].

Assuming an illness rate of 10% and precision of 0.02, a sample size of 864 households was arrived at which was inflated to 1000 to account for the design effect of 1.15. Multistage cluster random sampling design was adopted. The number of clusters was fixed at 28 and to achieve a final sample size of at least 1000 households, 36 households were selected from each cluster as follows.

Assuming hospitalization rates to be at least 1% (rarest event), 300 households (approximately 1500 persons) were to be screened in each cluster to prepare lists of following four types of households, and from these lists 36 households were to be randomly sampled as follows: (a) hospitalization in the last one year (15 households), (b) treatment sought for illness lasting for more than 30 days (7 households), (c) treatment sought for illness in the last 30 days (7 households), and (d) currently pregnant women in the household or a woman who had been pregnant in the last two years, irrespective of the outcome (7 households). If in the sampled household, there were multiple type of cases as defined above all types of cases were to be included in the sample.

In the first stage, clusters were categorized into rural and urban, in proportion to population size using Census of India 2011 sample frame. A village and a municipal ward formed a cluster in rural and urban areas respectively. Eighteen clusters were allocated to rural and 10 to urban strata. In second stage, stratification of clusters was done based on population in each cluster: strata I with population <999, strata II with population 1000–1999, strata III with population 2000–4999, and strata IV with population > 5000. Selection of villages and wards in various strata were as follows- strata I: 5 villages and 2 urban wards, strata II: 4 villages and 2 urban wards, strata III: 3 villages and 3 urban wards, strata IV: 6 villages and 3 urban wards.

In third stage, in each of the randomly selected villages and wards, 300 households were screened consecutively after choosing a random starting point. If the village/ ward had <300 households then all households were included. A structured household questionnaire was used to collect socio-demographic details, household expenditure, type of illnesses, health seeking behavior, and expenditure on health for the cases mentioned above. Written informed consent was obtained from the participants for administering questionnaire. The survey had approval of the Institute Ethics Committee. Handheld tablet computers were used for data collection, storage and transmission through wireless network. Center for Development of Advanced Computing (CDAC), Mohali, Punjab developed the software application based on the questionnaires designed by National Health System Resource Center, New Delhi. The computer application included auto-generated options and logical question flows (skip rules and validation checks) to ensure complete data collection. Seven field investigators and two supervisors with graduate level education were trained in data collection to maintain quality. All field investigators were comfortable in using tablet computers. Data collection was slow initially but gained momentum as the enumerators got used to the skips and check pattern in the questionnaire. Each interview lasted about 20–25 minutes. The entire activity was completed in 2 months.

### Statistical analysis

Data was captured on the computer server using Microsoft Excel Software. After checking for the range and consistency, descriptive analysis was done using SPSS (version 16.0, Illinois, Chicago, USA).

Impoverishment status of the household was assessed by enquiring about the enrolment in public distribution system for subsidized food rations by the government authorities. Household consumption expenditure was calculated by combining expenditure on food and non-food items including household items, fuel, personal transport, taxes and rents, health, education, clothing, social/ religious/ festival expenditure etc. Household consumption expenditures were expressed as monthly expenditures. Healthcare expenditure was calculated under separate heads, e. g, institutional charges or doctor’s fee, medicines, diagnostics, and transport etc.

Households were classified into quartiles based on monthly household consumption expenditure; quartile one representing the richest and quartile four representing the poorest households respectively. Catastrophic health expenditures (defined as >10% of the total household expenditure) [12] on hospitalization in the past one year was analysed according to the consumption expenditure quartiles. Chi square trend for trend was used to test the statistically significant differences.

## Results

A total of 1,010 households (consisting of 4227 individuals) were included in the study. Mean household size was 4.2. The mean age of the household members was 34.2 years (SD 19.5). Seventeen percent of the population was less than 15 years-old, 52.1% were between 15 to 45 years and 13.7% were more than 60 years. Fifty five percent were males. Nearly 80% were literate. Heads of households were laborers (23.9%), businessmen (13.9%), farmers (11.3%), employed in salaried jobs (12.2%), and 5% were pensioners or 23.1% were dependent on other members. Most (86.3%) of the households had pucca houses with tap water (88.9%) for drinking and 75% had facility of flush or sanitary latrine. Liquefied petroleum gas was the primary energy source for cooking (57%), followed by firewood (35%). About 22% of the household were below poverty line. Average monthly household expenditure was Indian Rupees (INR) 15,029 (USD 225). Average monthly expenditure on food, non-food articles and health was INR 5,038 (USD 75.4), INR 9,990 (USD 142) and INR 2,013 (USD 30.4) respectively.

Fever (25%), respiratory tract disease (14.9%), gastrointestinal diseases (14%) musculoskeletal disorders (13%) and skin diseases (6%) were the common acute illnesses, while heart disease (24%), diabetes (18%), respiratory disease (17%) and hypertension (15%) were the common chronic diseases (Table 1). Majority (83%) of persons sought healthcare for acute illness (83%) and chronic illness (78.6%) from private sector. Five persons (2.5%) were not visiting any healthcare provider despite the advice by the health care provider to do so as they continued taking medication as per previous prescription.

**Table 1:** Pattern of illnesses in Shaheed Bhagat Singh Nagar District of Punjab, India, 2014.

Illnesses*	Chronic Illnesses (N = 280) n (%)	Acute Illnesses (N = 188) n (%)	Hospitalizations (N = 426) n (%)
**Heart disease**	181 (24)	1 (0.5)	71 (16.7)
**Diabetes mellitus**	138 (18.3)	6 (3.2)	14 (3.3)
**Airway disease & asthma**	126 (16.7)	28 (14.9)	39 (9.2)
**Hypertension**	115 (15.3)	6 (3.2)	-
**Bone/joint disease**	82 (10.9)	25 (13.3)	20 (4.7)
**Skin diseases**	24 (3.2)	11 (5.9)	7 (1.6)
**Gastric/peptic ulcer/ GI tract**	26 (3.4)	27 (14.4)	68 (16)
**Neurological**	11 (1.5)	7 (3.7)	21 (4.9)
**Cancers/tumor**	10 (1.3)	-	14 (3.3)
**Liver disease**	9 (1.2)	-	24 (5.6)
**Mental illness**	9 (1.2)	3 (1.6)	-
**Tuberculosis**	7 (0.9)	-	3 (0.7)
**Ocular/ENT**	5 (0.7)	-	18 (4.2)
**Epilepsy**	4 (0.5)	-	-
**Genitourinary**	4 (0.5)	-	19 (4.5)
**Endocrine (thyroid)**	3 (0.4)	-	-
**Fever**	-	33 (17.5)	1 (0.24)
**Others (including fever)**	-	41 (21.8)	107 (25.3)

*Many people had multiple illnesses

Hospitalizations were due to cardiovascular diseases (15%), gastrointestinal problems (14%), accidents (12%), respiratory problems (9%) and infectious diseases (8%) (Table 1).

Only about 20% of the hospitalizations were availed in government facilities. Among government facilities, districts hospitals (7.5%) followed by medical colleges (6.8%) were frequently availed. Average duration of hospitalization was 5 days; 26.1% were hospitalized for 3 days or less, 54.9% were hospitalized for 4–10 days and 1.4% for more than 30 days.

The average monthly expenditure on acute illness was INR 806 (USD 12) and for chronic illness it was INR 1,795 (USD 27) per month. Expenditure on acute illness was met from current income (81.3%) followed by past saving (18.6%). Expenditure on chronic illness came mainly from current income (67.9%), followed by past savings (29.3%). Reimbursement from insurance and other sources was in only less than 1% cases (Table 2).

**Table 2:** Sources of expenditure for health conditions in Shaheed Bhagat Singh District of Punjab, India, 2014.

Sources#	Chronic illnesses (N = 280) n (%)	Acute illnesses (N = 188) n (%)	Hospitalizations (N = 426) n (%)	Childbirth in last 2 years (N = 120) n (%)
**Current income**	190 (67.9)	153 (81.4)	107 (25.1)	74 (61.6)
**Past savings**	82 (29.3)	35 (18.6)	237 (55.6)	63 (52.5)
Sale of assets*	4 (1.4)	-	18 (4.2)	-
**Donation by friends & relatives**	15 (5.4)	1 (0.5)	184 (43.2)	22 (18.3)
**Borrowings through mortgage/ loans**	5 (1.8)	-	52 (12.2)	11 (9.2)
**Re-imbursements (insurance/ employer/ government scheme)**	1(0.4)	-	3 (0.7)	72 (60)
**Other sources**	1(0.4)	-	1 (0.2)	-

^#^ Multiple response

*ornaments /land/ grains/ livestock

The mean expenditure incurred on hospitalization was INR. 43040 (USD 644). The major reason of expenditure was on purchase of medicines/ consumables and diagnostic tests. The common source for meeting hospitalization expenses was past savings (55.6%), borrowing from friends and relatives (43.1%), current income (25.1%), borrowing through mortgage or loans (12.2%), and sale of assets (4.2%). Less than one percent was through reimbursement or insurance or other schemes such as *Rashtriya Swasthya Bima Yojana* (RSBY) (Table 3).

**Table 3:** Type of healthcare expenditure in Shaheed Bhagat Singh District of Punjab, India, 2014.

Type of Expenditure	Mean Expenditure in Indian Rupees (USD)*					
	N	Acute illnesses	N	Chronic illnesses	N	Hospitalizations
**Consultation/ Service fee**	180	101.4 (1.5)	213	104 (1.5)	419	18294 (270.7)
**Diagnostic tests (lab & radiology)**	163	537.4 (8.0)	117	775 (11.5)	386	6258 (92.6)
**Medicines and consumables**	163	386.4 (5.7)	213	1101 (16.3)	385	12479.8 (184.7)
**Transportation**	139	78.7 (1.2)	213	170 (2.52)	376	876 (12.9)
**Informal payments**	5	129 (1.9)	7	695 (10.3)	14	6157 (91.1)
**Total**		806 (11.9)		1795 (26.7)		43040(636.9)

*USD = United States Dollars

Expenditure in government health facilities was less than in private health facilities for all events included in the study which required healthcare (Table 4).

**Table 4:** Healthcare expenditure in government and private health facilities in Shaheed Bhagat Singh District of Punjab, India, 2014.

Reason for seeking health care	Mean Expenditure in Indian Rupees (USD)*	
	Government sector	Private sector
**Acute illness**	504 (7.5)	1019 (15.1)
**Chronic illness**	1040 (15.4)	2328 (34.5)
**Hospitalization**	23478 (347.4)	67345 (996.5)
**Live birth**	11117.5 (164.5)	22305 (334.2)
**Still birth**	14895 (220.4)	22585 (346.5)
**Normal delivery**	10895 (161.2)	22170 (328)
**Cesarean section delivery**	17475 (258.6)	22670 (335.5)

*USD = United States Dollars

Among the study households, 67 women were currently pregnant. Though all of the current pregnancies were registered with the Auxiliary Nurse Midwives (ANMs) appointed by the government, nearly 70% of pregnant women were also seeking antenatal services from the private sector. There were 75 recorded abortions; government health facility was visited in 30.6% of the abortions, and private health facility in 29.3%, and in 40% of the abortions no healthcare was sought.

One hundred and twenty women had experienced childbirth in the last two years. Almost all, 118 (98.3%) deliveries, had occurred in health institutions; 37 (31%) deliveries were in government health institutions. There were 96 (80%) live births and 24 (20%) intra-uterine deaths/stillbirths. Caesarean section was conducted in 22.5% of the deliveries. Caesarean section rate was 20% in government and 26% in private health facilities.

The mean expenditure on live births and stillbirths was INR 16,851 (USD 254.4) and INR 18,740 (USD 283) respectively. The mean expenditure for a normal delivery was INR 16,532 (USD 250), and for cesarean section delivery it was INR 20,072 (USD 303). Expenditures in private sector were higher than in the government sector (Table 4). About 60% of the women had received financial benefits under the *Janani Suraksha Yojana* and *Mata Kaushalya Scheme* run by Punjab Government to promote institutional deliveries.

There was non significant difference in the rate of hospitalization between the richer and the poorer quartiles. Proportion of households experiencing catastrophic expenditures on hospitalization was 55% in the richest and 75% in the poorest quartile (p<0.002). There was a significant increasing trend of catastrophic expenditure from richest to the poorest quartile (Table 5).

**Table 5:** Catastrophic expenditure amongst hospitalized cases in Shaheed Bhagat Singh District of Punjab, India, 2014.

Expenditure Quartile	Hospitalized N	Households incurring catastrophic expenditure* n (%)
**Q1 (Richest)**	96	53 (55.2)
**Q2**	116	60 (51.7)
**Q3**	104	62 (59.6)
**Q4 (Poorest)**	110	82 (74.5)
**Overall**	426	241 (56.5)

* χ^2^ for linear trend = 9.8, p <0.002

The onetime cost of using this survey methodology was almost 70% of the estimated expenditure that would have been incurred had we used the traditional paper based surveys. Estimated cost of making requisite paper copies of questionnaires and dual data entry was about INR 2,55,000 (USD 3764). The cost of tablets used was INR 8500 (USD 128) per unit. Cost of software development was INR 1,00,000 (USD 1510). None of the tablets was damaged or lost during the survey. Visibility of the LED screen of tablets was poor in the sunlight. Additionally, the heated environment didn’t allow the tablets to cool and the tablets shut down occasionally. Internet connectivity was intermittent. The enumerators could only upload data after returning to the main city where connectivity was available. The battery did get discharged quicker due to the heat. The working time available with each battery charge was about 3.5–4 hours. On cooler days, battery outlasted the working hours.

## Discussion

This rapid survey reveals that dual burden of communicable and non-communicable diseases exist in state of Punjab where healthcare is sought more often from the private sector and out-of-pocket expenditures are high. A high proportion of households having an episode of hospitalization experienced catastrophic expenditure with the poorer sections experiencing higher rates of improvishment. A study done in Bengal in 2007 reported that the annual per household expenditure on OPD visits, chronic diseases, and hospitalizations was INR 1170 (USD 17.7), INR 2637 (USD 40), and INR 4340 (USD 65.5), respectively[13]. The expenses of hospitalization were also higher in our study than the NSSO estimates[5] and the Bengal study [13]. The out-of-pocket expenditure on hospitalization was higher too. Some of the rise in expenditures could be due to inflation or price rise as our study was conducted in 2014 as against the above mentioned studies which were done earlier.

The 2004 NSSO report states that nearly 73% of healthcare financing is through current income^5^. High out-of-pocket healthcare expenditure in Bengal resulted in 34% of households losing all their past savings, 30% households borrowing on interest and 2% household selling assets[13]. Peters (2002) reported that nearly 40% of hospitalized patients had to sell assets or borrow money to pay for hospital costs in India [14]. Duggal (2004) reported that among the poorest quintile in India, 50% respondents had to pay from savings or borrowings for healthcare expenditure [15]. In a study in rural Orissa, about 25% of households reported hardship in financing healthcare. Amongst the household facing hospitalization, 40% reported hardship [16]. In our study 56.5% of the households whose member was hospitalized faced catastrophic expenditure with the poorest being the worst affected. Nearly 12.2% had to borrow on interest and 4.2% resorted to sale of assets. The scenario may be worse than this because there is high likelihood that the poorest quartile does not seek health care due to financial hardship as has been reported in previous rounds of NSSO 2004 [2]. For achieving UHC, measures to reduce high out-of-pocket expenditures need to be adopted. In our study nearly 15% of the respondents had utilized the public sector for outpatient healthcare visits and hospitalization which was similar to 19.9% reported by a study done in Haryana [17]. This shows the people prefer private facilities over public healthcare facilities in Punjab also. Public sector has been perceived as technically competent but inconvenient and provider centered, with complex systems that take time and effort to negotiate [18]. Other studies in India report that patients are examined for longer duration, were more likely to have a physical examination during visit, and diagnosis explained by private sector physicians compared to those in public sector [19].

Chaudhuri pointed out that despite an increased utilization of health services by the poor, hospitalization and outpatient services at public health facilities are still very low in Haryana (30%) and Punjab (20%) [20]. Another study from north India found that use of public hospitals was low and catastrophic expenditures were higher among the poor in Punjab and Haryana [21]. Study by Khumukcham *et al* (2015) also indicates preference for public health facility by lower socio-demographic individuals [22]. Findings by Dey and Mishra (2014) reveal that people with increasing age, females, lower income group people, uneducated and weaker sections of society are more likely to prefer public healthcare services as compared to private services in India [23]. A study by Chandra *et al* (2013) revealed that in 2004, nearly 68% of households were utilizing private health facilities [24].

However, a study from southern India shows that, in an area where health services are available free from the public sector, 75.5% of the households sought healthcare from the public health facilities and majority of the households (58.1%) did not incur any expenditure on healthcare at all. Most of the households which did incur expenditure on health had sought care from private sector and were from the higher socioeconomic strata [25] Kumar and Prakash (2011) point out that utilization of public health care services depends mainly on the provision of government-subsidized services. The utilization of public services appeared to be most likely driven by a desire to use government programs that provided free services or distribution of medicines, contraceptives or immunizations [26]. Medicines have been found to account for a very high proportion of hospital and outpatient expenditures in the public sector in Punjab, Haryana and Chandigarh [21]. Ghosh (2011) also found nearly 67% of total healthcare expenditure was on medicines [27]. Model based estimates also suggest that most of the out-of-pocket healthcare expenditure is on medicines and that provision of free medicines shall increase uptake of health services [28]. Our study too, found that medicines, consumables and diagnostics bear the maximum share of health expenditures. Policies targeted to reduce expenditures on these components may bring down out of pocket expenditure and will be a positive step towards achieving UHC.

Much higher institutional delivery rates were observed in this study in contrast to the DLHS-4 data. Institutional deliveries have shown a rising trend since the institution of special measures under National Rural Health Mission (NRHM) like *Janani Suraksha Yojna* and the deployment of Accredited Social Health Activists (ASHAs) in the villages. About 60% of the women derived such benefits in our study. These rates are much higher than the 17% reported in DLHS-4 [5].

The survey was done for SBS Nagar District of Punjab. Although SBS Nagar is a typical district of Punjab, caution has to be taken during generalizing the finding to the entire state due to small sample size. Secondly, the expenditure estimates were based on recall of participants. Such methods generally underestimate the expenditures but are useful for comparison and ranking purposes. Literature reveals similar observations by others also [29]. Thirdly, the morbidity pattern for last 30 days may change with season and study conducted in a different time frame may report different patterns. Self-reported morbidity may suffer from ‘positional objectivity’, *i*.*e*., the ‘position’ of the individual matters (in terms of education, income, *etc*.) in responding to questions related to self report [30]. However, large scale surveys have used this methodology and established it as a valid measure to estimate illness in a population [31].

The primary advantage of the survey was the rapid completion and the timeliness of the analysis. Rapid surveys to assess the health status of the population can contribute to better allocation of resources tailored to the health needs specific to the area and in turn institute policies that improve equity and reduce household spending on health. Besides data collected using Computer Assisted Personal Interviewing (CAPI) on tablet ensured better quality with fewer inconsistencies and missing cases, saved data entry time, and reduced the scope of field editing. The software and the tablets can be retained for repeated use in periodic surveys to assess universal healthcare coverage. Hence, the cost gains will accrue when repeat surveys are conducted. A major gain was on the time saved on dual data entry. Use of IT enabled data to be immediately available for analysis. In large scale surveys, this would be a huge advantage as results could be available almost immediately after data collection had been completed.

Problems did occur, during the course of the survey, such as hardware malfunction, poor internet connectivity, reluctance of some investigators to use computers for data entry. As the use of computing devices becomes more routine, it is expected that the healthcare workforce shall increasingly use computing devices and smart phones in their day to day work. However, the problem of battery dying out in short period or the device shutting down due to overheating in summer months does pose a logistical problem. Hardware innovations especially to improve visibility of the LED screens in sunlight should improve usability.

In conclusion, this rapid survey indicates that universal healthcare coverage is yet to be achieved in the district. Though healthcare facilities are available but government health facilities which are less expensive were utilized less often. Out-of-pocket expenditure was high and most households experiencing an episode of hospitalization underwent catastrophic expenditure, especially the more improvished. Most of the expenditure was on medicines and diagnostic services. Policies need to be adopted to ensure low cost diagnostic services and uninterrupted supply of medication through the public health system to increase utilization of government health service and to reduce out of pocket expenditure on healthcare as has been seen with the increased institutional deliveries which possibly have been encouraged by availability of incentives. Targeted preventive and curative measures of identified common morbidities in the community will help in achieving the universal health coverage.

## References

[1] Campbell J, Buchan J, Cometto G, David B, Dussault G, Fogstad H, et al Human resources for health and universal health coverage: fostering equity and effective coverage. Bull World Health Organ. 2013;91(11):853–63. 10.2471/BLT.13.118729 24347710 PMC3853950

[2] National Sample Survey Organisation. Level and pattern of consumer expenditure –61st round, 2004–05. New Delhi: Ministry of Statistics and Programme Implementation, Government of India; 2006

[3] International Institute for Population Sciences (IIPS) and Macro International. National Family Health Survey (NFHS-3), India, 2005–06: Punjab Mumbai: IIPS; 2008.

[4] Global Adult Tobacco Survey (GATS) India, 2009–2010. [Last cited on 2015 July 23]. Available from: http://www.mohfw.nic.in/WriteReadData/l892s/1455618937GATS%20India.pdf

[5] International Institute for Population Sciences (IIPS). District Level Household and Facility Survey (DLHS-3), 20012–13: India Mumbai: IIPS; 2013 http://www.rchiips.org/pdf/dlhs4/report/PJ.pdf [last accessed 2015 August 20]

[6] Henderson RH, Sundaresan T. Cluster sampling to assess immunization coverage: a review of experience with a simplified sampling method. Bull World Health Organ. 1982; 60: 253–60. 6980735 PMC2535957

[7] Leisher C. A comparison of tablet-based and paper-based survey data collection in conservation projects. Soc. Sci. 2014;3:264–271

[8] Paudel D, Ahmed M, Pradhan A, Dangol RL. Successful use of tablet personal computers and wireless technologies for the 2011 Nepal Demographic and Health Survey. Glob Health Sci Pract. 2013;1(2):277–284. 10.9745/GHSP-D-12-00056 25276539 PMC4168579

[9] https://nutritionrapidsms.wordpress.com/2014/06/05/nigeria-completes-national-nutrition-and-health-survey-with-data-collection-on-mobile-devices/ [last accessed 2015 August 1]

[10] Census of India 2011 Office of the Registrar General of India and Census Commissioner. New Dehi: Ministry of Home Affairs, Government of India.

[11] International Institute for Population Sciences (IIPS), District Level Household and Facility Survey (DLHS-4). District fact sheet: Shaheed Bhagat Singh Nagar 2012–13: Ministry of Health and Family Welfare: Mumbai: IIPS 2015.

[12] Xu K, Klavus J, Kawabata K, Evans DB, Hanvoravongchai P, Ortiz de Iturbide JP, et al Household health system contributions and capacity to pay: definitional, empirical and technical challenges In: Murray CJL, Evans DB, eds. Health systems performance assessment: debates, methods and empiricism. Geneva: World Health Organization; 2003.

[13] Mondal S, Kanjilal B, Peters DH, Lucas H. Catastrophic out-of-pocket payment for health care and its impact on households: Experience from West Bengal, India. Future Health Systems: Innovations for equity. 2010. [Last accessed on 2015 July 23]. Available from: http://www.chronicpoverty.org/uploads/publication_files/mondal_et_al_health.pdf

[14] Peters DH, Yazbeck AS, Sharma RR, Ramana GNV, Pritchett LH, Wagstaff A. 2002 Better Health Systems for India's Poor: Findings, Analysis, and Options. Washington, DC: World Bank © World Bank. https://openknowledge.worldbank.org/handle/10986/14080 License: CC BY 3.0 IGO.

[15] Duggal R. Financing healthcare in India-Prospects for health insurance. Express Healthcare Management. 2004. [Last accessed on 2015 July 23]. Available on http://www.cehat.org/go/HealthServicesandFinancing.

[16] Binnendijk E, Koren R, Dror DM. Hardship financing of healthcare among rural poor in Orissa, India. BMC Health Serv Res. 2012; 12: 23 10.1186/1472-6963-12-23 22284934 PMC3317855

[17] Ray TK, Pandav CS, Anand K, Kapoor SK, Dwivedi SN. Out-of-pocket expenditure on healthcare in a north Indian village. Natl Med J India. 2002;15:257–60. 12502135

[18] Berendes S, Heywood P, Oliver S, Garner P. Quality of private and public ambulatory health care in low and middle income countries: systematic review of comparative studies. PLoS Med. 2011;8(4):e1000433 10.1371/journal.pmed.1000433 21532746 PMC3075233

[19] Bhatia J, Cleland J. Health care of female outpatients in south-central India: comparing public and private sector provision. Health Policy Plan. 2004;19:402–409. 15459165 10.1093/heapol/czh055

[20] Chaudhuri A. Socio-economic inequity in health care utilization & expenditures in richer States in India. Indian J Med Res. 2012;136 (3):368–69. 23041729 PMC3510882

[21] Prinja S, Kanavos P, Kumar R. Health care inequalities in north India: Role of public sector in universalizing health care. Indian J Med Res. 2012;136:421–31. 23041735 PMC3510888

[22] Khumukcham T., Singh T., Kaur M., Gupta M., Kumar R. Factors influencing the choice of a public or private health institution for childbirth in Chandigarh. Indian J Comm Health. 2015;27(1):86–94.

[23] Dey DK, Mishra V. Determinants of choice of healthcare services utilization: empirical evidence from India. Ind J Comm Health. 2014;26(4):356–363.

[24] Chandra R, Singh A, Mukherjee S. A disaggregated analysis of change in household out-of-pocket expenditure on healthcare in India, 1995–2004. International Journal of Public Health Research.2013;3(1):257–66.

[25] Archana R, Kar SS, Premarajan KC, Lakshminarayanan S. Out of pocket expenditure among the households of a rural area in Puducherry, South India. J Nat Sci Biol Med. 2014 Jan-Jun; 5(1): 135–138. 10.4103/0976-9668.127312 24678212 PMC3961918

[26] Kumar C, Prakash R. Public-private dichotomy in utilization of health care services in India. The Journal of Sustainable Development. 2011;5(1): 25–52

[27] Ghosh S. Catastrophic payments and impoverishment due to out-of-pocket health spending. Econ Pol Wkly. 2011;46(47):63–70.

[28] Prinja S, Bahuguna P, Pinto AD, Sharma A, Bharaj G, Kumar V, et al The cost of universal health care in India: a model-based estimate. PLoS One 2012; 7: e30362 10.1371/journal.pone.0030362 22299038 PMC3267714

[29] Lu Chunling, Chin Brian, Li Guohong, Christopher JL Murray. Limitations of methods for measuring out-of-pocket and catastrophic private health expenditures. Bulletin of the World Health Organization. 2009; 87: 238–244. 19377721 10.2471/BLT.08.054379 PMC2654642

[30] Sen A. Positional objectivity. Philos Public Affairs. 1993; 22:126–45.

[31] Idler EL, Benyamini Y. Self-rated health and mortality: a review of twenty-seven community studies. J Health Soc Behav. 1997; 38:21–37. 9097506

