# Correction: Assessment of Universal Healthcare Coverage in a District of North India: A Rapid Cross-Sectional Survey Using Tablet Computers

**DOI:** 10.1371/journal.pone.0176063

**Published:** 2017-04-17

**Authors:** Tarundeep Singh, Pritam Roy, Limalemla Jamir, Saurav Gupta, Navpreet Kaur, D. K. Jain, K. Rahul Reddy, Tushar Mokashi, Rajesh Kumar

K. Rahul Reddy and Tushar Mokashi are not included in the author byline.

K. Rahul Reddy should be the seventh author and affiliated with Healthcare Financing Division, National Health Systems Resource Centre, Ministry of Health and Family Welfare, Government of India. The contributions of the author are as follows: conceived and designed the experiments

Tushar Mokashi should be the eighth author and affiliated with Healthcare Financing Division, National Health Systems Resource Centre, Ministry of Health and Family Welfare, Government of India. The contributions of the author are as follows: conceived and designed the experiments

The following information is missing from the Methodology section: The methodology was adopted from measurement of district level health financing indicators for Universal Health Coverage tool kit designed by National Health System Resource Center, New Delhi (Sundararaman et al. 2014). The reference is: Sundararaman T, Vidyanathan G, Vaishnavi SD, Reddy KR, Mokashi T, Sharma J et al. Measuring Progress towards Universal Health Coverage—An Approach in the Indian Context. Econ Polit Wkly. 2014 Nov 22; XLIX(47):60–65

Some information is omitted from the Acknowledgements section. The Acknowledgements should read: We are grateful to National Rural Health Mission Punjab for providing financial support for conducting this survey, and National Health System Resource Center, New Delhi for supporting us with research design, sampling, providing the questionnaire and training of investigators.
